# Hemophagocytic lymphohistiocytosis after SARS-CoV-2 vaccination

**DOI:** 10.1007/s15010-022-01786-y

**Published:** 2022-02-26

**Authors:** Marie-Lisa Hieber, Rosanne Sprute, Dennis A. Eichenauer, Michael Hallek, Ron D. Jachimowicz

**Affiliations:** 1 Department I of Internal Medicine, Center for Integrated Oncology Aachen Bonn Cologne and Duesseldorf, Faculty of Medicine, University Hospital Cologne, University of Cologne, Cologne, Germany; 2 Cologne Excellence Cluster on Cellular Stress Response in Aging-Associated Diseases, University of Cologne, Cologne, Germany; 3 German Centre for Infection Research (DZIF), Partner Site Bonn-Cologne, Cologne, Germany; 4 Max Planck Institute for Biology of Ageing, Cologne, Germany; 5 Center for Molecular Medicine Cologne, University of Cologne, Cologne, Germany

**Keywords:** Hyperinflammation, Hyperferritinemia, Kineret, Lymphadenopathy, Macrophage activation syndrome, SARS-CoV-2 vaccine

## Abstract

**Purpose:**

The coronavirus disease 2019 (COVID-19) pandemic has led to the approval of novel vaccines with different mechanisms of action. Until now, more than 4.7 billion persons have been vaccinated around the world, and adverse effects not observed in pre-authorization trials are being reported at low frequency.

**Methods:**

We report a case of severe hemophagocytic lymphohistiocytosis (HLH) after SARS-CoV-2 immunization and performed a literature search for all reported cases of COVID-19 vaccine-associated HLH.

**Results:**

A 24-year-old female developed HLH after immunization with the mRNA COVID-19 vaccine Comirnaty. Diagnosis was made according to HLH-2004 criteria; the HScore was 259 (> 99% HLH probability) with maximum ferritin of 138.244 µg/L. The patient was initially treated with intravenous immunoglobulins (IVIGs) and dexamethasone without response. The addition of the human interleukin 1 receptor antagonist Anakinra resulted in full recovery within 6 weeks after vaccination.

A literature search revealed 15 additional cases of HLH after SARS-CoV-2 vaccination, the majority after immunization with Comirnaty (*n* = 7) or the viral vector vaccine Vaxzevria (*n* = 6). Treatment modalities included corticosteroids (*n* = 13), Anakinra (*n* = 5), IVIGs (*n* = 5), and etoposide (*n* = 2). Eight patients underwent combination treatment. Three of 16 patients died.

**Conclusion:**

COVID-19 vaccines may occasionally trigger HLH, and Anakinra may be an efficacious treatment option for this condition.

## Introduction

Several vaccines with a different mechanism of action were developed to combat the recent pandemic caused by the severe acute respiratory syndrome coronavirus type 2 (SARS-CoV-2). Rare hematologic side effects are emerging with the increased use of these vaccines. Immune thrombocytopenia is the most frequently reported adverse hematologic effect after mRNA- and viral vector vaccines against coronavirus disease 2019 (COVID-19) [1, 2]. Here, we report a case of a severe hyperinflammation syndrome fulfilling the diagnostic criteria for hemophagocytic lymphohistiocytosis (HLH) after immunization with the mRNA COVID-19 vaccine Comirnaty (BNT162b2, Pfizer-BioNTech). We additionally summarize previously identified HLH cases following COVID-19 vaccination and highlight the possible role of a new treatment approach—the additional use of human interleukin 1 receptor antagonist—to improve outcomes of HLH patients.

## Methods

A literature search was performed in PubMed for all reported cases of COVID-19 vaccine-associated HLH since database inception until January 18, 2022. The predefined search filter ‘(HLH OR hemophagocytic lymphohistiocytosis OR haemophagocytic lymphohistiocytosis) AND (COVID-19 OR SARS-CoV-2) AND (vaccine OR vaccination)’ yielded 15 results. Reference lists of articles were screened for other suitable studies and authors were contacted to obtain additional data.

## Results and discussion

**Fig. 1:**
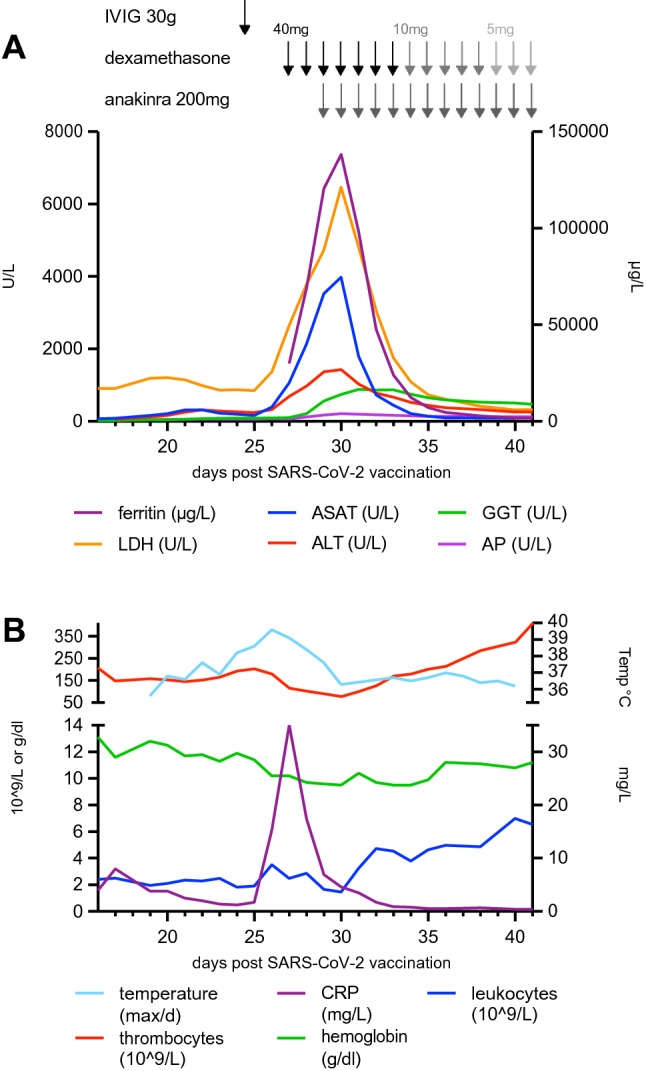
Changes in body temperature and laboratory parameters over the clinical course. Therapeutic approaches are marked with arrows. *ALT* alanine transaminase, *AP* alkaline phosphatase, *AST* aspartate transaminase, *CRP* C-reactive protein, *GGT* gamma glutamyltransferase, *IVIG* intravenous immunoglobulins, *LDH* lactate dehydrogenase

HLH is a life-threatening hyperinflammatory syndrome caused by aberrantly activated macrophages and cytotoxic T cells. HLH can rapidly progress to multiple organ failure and, if untreated, is often fatal [3]. Even with current treatment options, it has a 50% lethality [4]. A 24-year-old, white female with no remarkable medical or travel history developed fever and unspecific fatigue for ten days after the first COVID-19 vaccination with Comirnaty. After a slight improvement of symptoms, she again developed fever, chills, increasing weakness, and nausea from day 13 after vaccination. At the time of presentation at the emergency department on day 16, laboratory testing revealed a reduced total white blood cell count (WBC, 2.4 × 10^9/l), elevated lactate dehydrogenase (LDH, 904 U/l), and slightly elevated aspartate aminotransferase (AST, 72 U/l). The patient was in a reduced general condition with a painful cervical and supraclavicular bilateral lymphadenopathy. Subsequent laboratory testing on day 19 showed a further decrease of the WBC count (1.95 × 10^9/l), an increase of LDH (1184 U/l) and liver function parameters (ASAT 162 U/l, ALAT 121 U/l, GGT 40 U/l), as well as decreased haptoglobin (< 0,1 g/l) (Fig. 1). Serum creatinine levels were normal at all times. Urinalysis was unremarkable. An abdominal ultrasound revealed a splenomegaly (158 × 57 mm), while a computed tomography chest scan confirmed enlarged cervical and supraclavicular lymph nodes with a maximum diameter of 19 × 10 mm. No additional lymphadenopathy or pulmonary infiltrates were detected. Serological and PCR virus tests (EBV, CMV, hepatitis B, C, E, HIV, HSV, Parvo-B19) were negative. The only remarkable value in the serological screening was an increase in *Mycoplasma pneumonia* IgG (22.3, reference < 9) and IgM (15.9, reference < 9) by enzyme immunoassay, though ten days later, no significant changes in antibody titers were observed. Coombs test, cold agglutinins, hemoglobin electrophoresis and glucose-6-phosphate dehydrogenase activity showed unremarkable results. In immunoblot, antinuclear antibodies (ANA) were positive. ANA differentiation detected antibodies against U1-RNP and PM-Scl. In the absence of other ANA and negative PM-Scl in a control measurement these findings were interpreted as an unspecific reaction. Testing for anti-double-stranded DNA and antineutrophil cytoplasmic autoantibodies (ANCA) was negative. Peripheral blood smears showed few large granular lymphocytes. A bone marrow aspirate and biopsy did not reveal further pathological findings, including no histological evidence for hemophagocytosis.

We diagnosed HLH based on the presence of five out of the eight HLH-2004 diagnostic criteria (Fig. 1 and Table 1). The HScore was 259 points (> 99% HLH probability) [5, 6]. During the workup of the patient, 30 g of intravenous immunoglobulins (IVIG) were administered on day 24 after vaccination but did not stop disease progression (Fig. 1). On day 27, dexamethasone 40 mg/d was initiated, but the patient had a steep increase in all HLH-relevant lab parameters including a maximum ferritin of 138.244 µ/l until day 30 and developed an acute liver failure (Fig. 1). In considering alternative treatment options, we reviewed the underlying mechanisms of vaccination-induced HLH. It has been shown that the SARS-CoV-2 spike protein induces IL-1β secretion in macrophages while the pro-inflammatory cytokine IL1-1β has an important role in hyperinflammation syndrome caused by COVID-19 [7]. The mRNA vaccine BNT162b1 encodes the SARS-CoV2 spike protein in full-length [8]. Thus, an IL-1β driven hyperinflammation syndrome after immune-stimulation by mRNA SARS-CoV-2 vaccination is likely a pathomechanism. Based on our understanding, we added the human interleukin 1 receptor antagonist Anakinra to the immunosuppressive treatment on day 29, given that it targets pro-inflammatory cytokine IL1 pathway. The patients’ general condition improved shortly thereafter and fever and abnormal laboratory findings gradually resolved. Dexamethasone was tapered from day 34 onwards, while Anakinra was administered beyond the patient’s discharge on day 41 (Fig. 1).

**Table 1:** Synopsis of clinical features of patients with hemophagocytic lymphohistiocytosis (HLH) after vaccination against SARS-CoV-2 infection described in literature

	Case	Vaccine	Symptom onset after vaccination	Medical history	HLH-2004 diagnostic criteria^a^	HScore	Treatment	Outcome
Our case	24 years, F	Comirnatry (BNT162b2 mRNA), Pfizer-BioNTech	10 days after 1st vaccination	No past medical history	1, 2, 3, 4, 5	259	IVIG, dexamethasone, Anakinra	Discharged 14 days after treatment initiation in good condition
Caocci et al. [14]	38 years, F	Comirnatry (BNT162b2 mRNA), Pfizer-BioNTech	21 days after 2nd vaccination	No past medical history	1, 4, 6, 7, 8	147	Methylprednisolone	Discharged after 1 week, fully recovered within weeks
Rocco et al. [13]	52 years, M	Comirnatry (BNT162b2 mRNA), Pfizer-BioNTech	1 day after 1st vaccination	Viral syndrome for 4 months, malaise	1, 2, 4, 5, 6, 8	239	Dexamethasone, etoposide	Death (neutropenic fever and *Bacteroides* bacteremia)
	53 years, M	Comirnatry (BNT162b2 mRNA), Pfizer-BioNTech	4 days after 1st vaccination	Interstitial lung disease	1, 4, 6	213	Dexamethasone, Anakinra, IVIG, rituximab	Ventilatory support for 3 months; discharged to rehab facility
	75 years, M	Spikevax (mRNA-1273), Moderna	12 days after 1st vaccination	Heart failure, HIV, Mycobacterium avium, KSHV viremia	3, 4, 5	185	Methylprednisolone	Death (encephalopathy and shock)
	55 years, F	Comirnatry (BNT162b2 mRNA), Pfizer-BioNTech	3 days after 1st vaccination	MAC, pulmonary aspergillosis, MDS	1, 2, 3, 4	208	Anakinra	Slowly recovered
	48 years, F	Spikevax (mRNA-1273), Moderna	4 days after 1st vaccination	HIV disseminated MAC and IRIS	1	130	Prednisone, infliximab	Improvement within 72 h
Cory et al. [15]	36 years, F	Vaxzevria (ChAdOx1), AstraZeneca	9 days after 1st vaccination	No past medical history	1, 2, 4	200	Methylprednisolone, IVIG	Improvement within 72 h, 2nd episode after 6 days, improved after IVIG
Baek et al. [16]	20 years, M	Comirnatry (BNT162b2 mRNA), Pfizer-BioNTech	2 days after 1st vaccination	No past medical history	1, 2, 4, 6, 7, 8	229	Dexamethasone	Immediate improvement
	71 years, F	Vaxzevria (ChAdOx1), AstraZeneca	7 days after 1st vaccination	Hypertension	1, 2, 4, 5, 6, 8	293	Dexamethasone, etoposide	Discharged after 8 weeks in good condition
Tang et al. [11]	43 years, F	Chinese inactivated SARS-CoV-2 vaccine	Shortly after 1st vaccination	No past medical history	1, 3, 4, 5, 6, 7	261	Dexamethasone	Discharged 17 days after start of dexamethasone
Ai et al. [12]	68 years, M	Vaxzevria (ChAdOx1), AstraZeneca	10 days after 1st vaccination	Hypertension, gout, Bowen’s disease	1, 2, 4, 6	250	No therapy	Spontaneous improvement
Sassi et al. [10]	85 years, M	Comirnatry (BNT162b2 mRNA), Pfizer-BioNTech	Shortly after 1st vaccination	No past medical history	6	Not calculated	No information	No information
Attwell et al. [9]	~ 65 years, M	Vaxzevria (ChAdOx1), AstraZeneca	5 days after 1st vaccination	Diabetes mellitus type II	1, 4, 5, 6, 8	259	Methylprednisolone, IVIG, Anakinra	ICU care, CVVH, vasopressor treatment, rapid biochemical improvement
	~ 75 years, F	Vaxzevria (ChAdOx1), AstraZeneca	7 days after 1st vaccination	Essential thrombocythemia, breast cancer in remission, bee sting anaphylaxis	1, 4, 5, 6, 8	220	Methylprednisolone, IVIG, Anakinra	ICU care, vasopressor treatment, rupture of the esophagus, died
	~ 35 years, M	Vaxzevria (ChAdOx1), AstraZeneca	8 days after 1st vaccination	Ankylosing spondylitis	1, 2, 4, 6, 8	219	Methylprednisolone	Good response

^a^HLH-2004 diagnostic criteria [3]: 1—fever (≥ 38.3 °C); 2—splenomegaly; 3—cytopenias in ≥ 2 lines (hb < 9 g/dL, plt < 100/nL, neutrophils < 1.0/nL); 4—ferritin ≥ 500 μg/L; 5—hypertriglyceridemia and/or hypofibrinogenemia (fasting triglycerides ≥ 265 mg/dL, fibrinogen < 1.5 g/L); 6—hemophagocytosis in bone marrow or spleen or lymph nodes; 7—low or absent NK activity; 8—soluble CD25 (soluble IL-2 receptor) ≥ 2400 U/mL

Until now, 16 cases of HLH after COVID-19 vaccination have been described (Table 1) [9–16]. Patients with and without pre-existing conditions and from all age groups (range 20–85 years) were affected. 8/16 cases were female. Seven patients developed HLH after immunization with Comirnaty, six after Vaxzevria (AstraZeneca), two after Spikevax (Moderna) and one after an inactivated SARS-CoV-2 vaccine. Symptoms occurred on average 7.4 days after vaccination (*n* = 14). 13/15 patients received corticosteroid treatment and 5/15 patients were treated with Anakinra and IVIGs, respectively. Etoposide was used in 2/15 patients. In 8/15 cases, combination therapy was administered. For one patient, treatment was not reported. 3/15 patients died despite appropriate initiation of treatment.

The observed duration between vaccination and onset of symptoms correlates with the upregulation of cytokine signature within days after COVID-19 vaccination [17] and is in line with other studies reporting duration of 10 days between diagnosis of underlying HLH trigger and occurrence of first symptoms [4]. In this case series, etoposide was the agent least frequently administered. The use of etoposide for immunosuppression in HLH according to the HLH-1994 protocol [18] is frequently limited by toxicity in patients with hepatic dysfunction. Comparisons of different HLH treatment strategies in adults with evidence from larger prospective studies are lacking, yet alternative strategies are becoming increasingly available. Anakinra has a good safety profile and a retrospective case series has shown clinical improvement and promising survival rates in combination with IVIGs or/and corticosteroids in patients with reactive HLH [19]. In addition, a favorable response to Anakinra treatment was reported in patients with COVID-19-associated HLH [20]. Anakinra has also been shown to significantly decrease mortality in COVID-19 patients with elevated soluble urokinase plasminogen activator receptor (suPAR) serum levels as a marker of pathogenic inflammation [21]. Based on our and other described reports (Table 1), as well as on a potential influence of pro-inflammatory cytokine IL1-1β [7], we suggest that patients diagnosed with HLH following a SARS-CoV-2 vaccination may benefit from the addition of Anakinra to the immunosuppressive treatment regimen for hyperinflammation syndrome. Moreover, the possibility of a SARS-CoV-2 vaccine-associated HLH should be kept in mind in the clinical routine to initiate early and targeted therapy.
